# Comparative Outcomes of Three School-Based Cognitive-Behavioral Interventions for Adolescent Suicide Prevention in Hong Kong

**DOI:** 10.3390/healthcare12202056

**Published:** 2024-10-16

**Authors:** Yiu Tsang Low, Kit Wai Lee

**Affiliations:** 1 Felizberta Lo Padilla Tong School of Social Sciences, Saint Francis University, Hong Kong, China; 2 Children and Youth Research Centre, Saint Francis University, Hong Kong, China; kw2lee@sfu.edu.hk

**Keywords:** suicide prevention program, adolescent, depression, suicide ideation

## Abstract

**Background/Objectives**: This study assessed the effectiveness of a school-based suicide prevention program in Hong Kong. **Methods**: 105 secondary school students aged 14–16, identified as having high levels of depression and suicidal ideation. Participants were divided into three intervention groups: one for adolescents only, another for adolescents with peers, and a third for adolescents with parental involvement. All groups engaged in weekly online exercises. Repeated measures ANOVA was used to analyze the within-group and between-group differences in the levels of adolescents’ suicidal ideation, depression and anxiety. **Results**: The results indicated a statistically significant reduction in suicidal ideation, depression and anxiety levels after participation in all three groups. No statistically significant between-group differences were identified, meaning the effects of the three interventions on the measured outcomes were similar. **Conclusions**: This study demonstrates the promise of implementing school-based suicide prevention programs in the Hong Kong context.

## 1. Introduction

Adolescent suicide is an alarming concern in Hong Kong. The Samaritan Befrienders Hong Kong reported 22 cases of youth suicide attempts or deaths between July and October 2023. Between September and October 2023, there were 14 attempts [1]. The suicide rate for 15–24-year-olds in Hong Kong rose from 6.1 deaths per 100,000 people in 2014 to 12.2 deaths per 100,000 people in 2022 [2]. Studies conducted in Hong Kong have shown significant rates of suicidal ideation among adolescents. One study found that 52.6% of Hong Kong Chinese adolescents aged 14–20 reported suicidal ideation [3]. A study on cyberbullying victimization among Hong Kong adolescents indicated that 21.8% reported suicidal ideation in the previous year [4]. Furthermore, lower self-esteem and depression among adolescents correlated significantly with adolescent suicide [5]. Additional factors such as childhood abuse, peer alienation, and hopelessness were positively related to depressive symptoms, which are significant predictors of suicidal ideation [6]. Stressful relationships between adolescents and their peers and families, feelings of loneliness and increased impulsivity are associated with adolescents’ suicidal ideation [7]. International studies have indicated that having a family history of suicide [8] and a personal history of attempted suicide are strong predictors of completed suicide. Poor family relationships, such as lower familial adaptability and family cohesion, are predictors of adolescent suicidal attempts [9]. Parent–adolescent conflicts are also a common factor in both completed and non-fatal suicidal episodes, and related to suicidal thoughts among college students [7,10]. Furthermore, a high number of conflicts between parents and adolescents, low cohesion within the family, ineffective parenting, including conflict-ridden and unsatisfying father-child relationships, and family discord and negative relationships with parents are also strong risk factors for youth suicide and depression [11]. Conversely, positive peer support is also important for adolescent mental wellbeing, which is an essential factor in suicidal ideation among adolescents. Supportive peer relationships at a time when an adolescent faces stressful situations also lower the risk of internalized and externalized symptoms [12]. Strong peer support negatively correlates with suicidal ideation [13].

### Preventive Programs Against Adolescent Suicide

In the context of adolescent suicide prevention, previous research has attempted to establish school-based interventions targeting suicide and associated risk factors among adolescents [14]. However, a significant gap exists in Hong Kong’s secondary education system, where a systematic and comprehensive suicide prevention program has yet to be implemented. The absence of a structured approach to both the delivery and evaluation of such programs in Hong Kong secondary schools is particularly noteworthy. Prior interventions have primarily focused on adolescents in isolation, neglecting the potential involvement of parents, educators, and peers [14]. A notable study in this field implemented a twelve-session program exclusively for adolescents within school settings, employing a cognitive-behavioral [15] approach to address key risk factors such as depression, self-esteem, problem-solving skills, and help-seeking behavior. This intervention demonstrated efficacy in enhancing participants’ help-seeking behavior and self-esteem, marking a significant step forward in the field of adolescent suicide prevention. A comprehensive review of international research on the efficacy of programs designed to enhance protective factors against adolescent suicide [16] revealed that the majority of school-based suicide prevention initiatives primarily focus on mitigating individual risk factors [17], training school personnel as gatekeepers [18], and implementing programmatic designs that incorporate educational elements alongside screening procedures for at-risk adolescents [19]. However, a significant limitation of many suicide prevention programs is their tendency to concentrate solely on at-risk adolescents, neglecting the potential involvement of family members or peers. A comparative study examining the efficacy of suicide prevention programs with parental inclusion demonstrated significantly superior outcomes in reducing hopelessness, anxiety, family dysfunction, and adolescent suicidal ideation while simultaneously enhancing adolescent personal self-control and family support [20]. Among the suicide prevention programs addressing various risk factors, anxiety and depression are frequently targeted [21]. Additionally, some programs aim to augment participants’ knowledge about suicide and focus on transforming undesirable attitudes into positive, help-seeking behaviors.

A review of previous studies on the outcome of suicide prevention programs in middle and high schools indicated significant improvements in participants’ knowledge, attitude and help-seeking behavior. Nevertheless, these programs have been less effective in reducing suicidal ideation and suicide rates [22]. As regards intervention theories found to be effective in delivering school-based prevention programs, CBT programs are the major type of intervention resulting in significant reductions in depressive symptoms [23]. Furthermore, prevention programs with homework assignments delivered by professionals are more effective than programs without such elements [24].

Previous research on suicide prevention programs has predominantly focused on individual risk factors, neglecting the broader psychosocial risk factors inherent in family and peer systems. The study reported in this article addressed this research gap through a comparative analysis of the efficacy of three distinct school-based adolescent suicide prevention programs: one involving adolescents with peer and teacher support, the second incorporating adolescents-plus-parent sessions, and the third consisting of parental programs supplemented with weekly online exercises for adolescents. This approach was informed by local studies demonstrating the efficacy of short-term parental interventions in reducing parent–adolescent conflict [25], a known risk factor for adolescent suicidal ideation. Furthermore, prior research found that programs incorporating parental involvement significantly improved outcomes related to adolescent mental health, including reductions in suicidal ideation and emotional distress [20], and some other programs highlighted the benefits of involving healthy peers as a form of support, which has been shown to enhance participants’ social support networks and, consequently, the psychological wellbeing of at-risk adolescents [26]. The proposed comparative analysis of these three intervention programs has the potential to enhance our understanding of optimal strategies for developing intervention programs specifically tailored to adolescents in Hong Kong based on local empirical data. This approach facilitates the customization and refinement of prevention programs using a ‘bottom-up’ methodology rooted in evidence-based practice.

## 2. Materials and Methods

This study utilized an experimental design to compare three different interventions. Pre- and post-intervention scores for the self-reported measures were analyzed. Due to ethical concerns regarding the provision of services to students in need, no control group was included in this study.

### 2.1. Recruitment and Participants Selection

The recruitment process for this study involved the distribution of invitation letters to various secondary schools in Hong Kong. A total of 15 secondary schools, supported by a collaborating non-governmental organization, were invited to participate in this project. Five secondary schools ultimately consented to participate in the research. The students’ backgrounds range from Band 1 to Band 3 schools (in Hong Kong, secondary schools are categorized into three bands based on the academic performance of the students they enroll). A total of 105 students were enrolled across three intervention groups, with schools randomly assigned to one of the interventions. The study targeted students aged 11–16 who completed a screening questionnaire assessing anxiety and depression levels. Students who scored high on the depression scale (≥9) were invited to participate in the intervention. Participation was contingent upon obtaining written parental consent and student assent. Students who exhibited elevated depression scores but declined to participate in the intervention were referred to school social workers for individual and family follow-up. Participants completed questionnaires assessing levels of depression, suicidal ideation, and parent–adolescent conflicts both prior to and following the intervention to evaluate the program’s outcome.

### 2.2. Three Treatment Programs

The first intervention group, designated as Group 1, comprised an adolescent-focused program supplemented with weekly online exercises or homework for four weeks following the group sessions. This intervention was structured as a six-week program, adapted from a locally developed depression prevention initiative [14]. The group content encompassed psychoeducational and skill-building components, including cognitive restructuring techniques, effective communication strategies, problem-solving methodologies, conflict resolution tactics, and anger management skills. Participants were provided with online weekly exercises after the completion of the six-week program to reinforce and extend the learning outcomes. This blended approach of in-person group sessions and digital follow-up activities aimed to enhance the retention and application of acquired skills, potentially optimizing the intervention’s outcome in addressing adolescent mental health concerns.

The second intervention group, designated as Group 2, incorporated a dual-component approach targeting both adolescents and their parents. The adolescent component was structured as a six-session intervention, followed by a four-session parental module focusing on positive parenting strategies and conflict resolution skills. Each session was delivered weekly. The parental component, adapted from previous research [25], integrated critical elements deemed essential for effective family-based interventions. By incorporating both adolescent-focused and parent-directed sessions, this intervention aimed to foster a synergistic effect, potentially enhancing the overall outcome of the program through the simultaneous development of adolescent coping skills and parental competencies.

The third intervention group, designated as Group 3, employed an approach extending beyond the core adolescent program to incorporate peer and teacher involvement. This intervention comprised the standard adolescent program, augmented by a four-week supplementary component integrating trained student leaders into the participant group. Prior to the commencement of the intervention, both teachers and student leaders undertook a specialized pre-group training session. This preparatory phase focused on enhancing their knowledge of adolescent suicide, depression, and community resources for suicide prevention. Additionally, the training emphasized the development of listening and communication skills. Following this preparation, the teachers and student leaders participated in four additional sessions alongside the adolescent participants.

### 2.3. Intervention Integrity

The implementation of the three intervention groups was conducted by two qualified social workers, each possessing three years of post-qualification experience and specialized training in CBT. The Principal Investigator (PI) conducted individual training sessions for all facilitators prior to the commencement of the interventions, focusing on comprehensive knowledge dissemination regarding the intervention protocols to ensure standardization and fidelity of program delivery. Throughout the intervention period, weekly individual meetings were held between the PI and each facilitator to maintain program fidelity and address any emerging concerns. Furthermore, the PI conducted direct observations of each group at three critical junctures: the beginning, middle, and end of each program. These observations were primarily aimed at assessing the facilitators’ skills and ensuring the quality and consistency of intervention delivery to the at-risk adolescent participants.

### 2.4. Instruments

Suicidal risk behavior. The Scale of Suicide Ideation (SSI), a 19-item, self-report [27], was adapted locally to quantify the intensity of current suicidal intent [5]. Each item consists of three alternative statements graded in intensity from zero to two. The total score ranges from zero to 38. The items assess the frequency and duration of suicidal thoughts. Previous studies suggest a cut-off point of four as the threshold for high risk of suicidal ideation [28]. Cronbach’s α for the reliability of the SSI in this study was 0.95, and construct validity was 0.92.

Depression. The Hospital Anxiety and Depression Scale (HADS-A) [29], includes fourteen items, seven of which comprise the anxiety subscale and seven the depression subscale. All items are scored on a four-point scale from zero to three. The Chinese version of HADS has been validated by Leung and colleagues [30], and was used in this study. The reliability of the overall scale was 0.86, and the reliability of the depression subscale was 0.82.

Parent–adolescent conflict. The Parent–Adolescent Conflict Scale (PAC) [31], is based on the Conflict Behaviour Questionnaire [32], and comprises 27 items measuring parent–adolescent conflict levels. PAC has been validated for use in Hong Kong by Shek and colleagues [33]. In this study, Chronbach’s α of the scale was 0.86, demonstrating high internal consistency and temporal stability.

### 2.5. Data Analysis

The analytical approach in this study adhered to the intention-to-treat principle, encompassing all recruited participants irrespective of their program compliance or participation levels [34]. A series of *t*-tests and chi-square tests were employed to identify any significant differences in demographic characteristics among the intervention groups to establish baseline equivalence. Additionally, a one-way analysis of variance (ANOVA) was conducted to assess potential pre-intervention differences across all measurement scales previously delineated. The primary analysis of treatment outcomes utilized a 2 × 2 mixed multivariate analysis of variance (MANOVA) with repeated measures (RM-ANOVA). In this analytical framework, the between-subjects factor was the group type (comprising the three distinct intervention groups), while the within-subjects factor encompassed the pre-intervention, post-intervention, and six-month follow-up assessments. This statistical approach enabled a comprehensive evaluation of the intervention effects across multiple time points and between different intervention modalities, thereby providing valuable insights into the comparative outcomes of the implemented suicide prevention programs.

## 3. Results

### 3.1. Differences Between the Three Intervention Groups

Table 1 shows there were no statistically significant differences between the three intervention groups. Table 2 indicates the comparison of five variables that we investigated during this study: anxiety level, depression level, anxiety and depression level, suicidal ideation, and parent–adolescent conflict level.

The findings of the correlation matrix (Table 2) reveal statistically significant positive correlations between anxiety and several other variables, including depression, suicidal ideation, and parent–adolescent conflicts. Similarly, depression demonstrates positive correlations with anxiety, suicidal ideation, and parent–adolescent conflicts. Conversely, social problem-solving skills are negatively correlated with suicidal ideation and parent–adolescent conflicts. These results corroborate our initial hypothesis, which posited positive associations between anxiety and depression with suicidal ideation and parent–adolescent conflicts. Furthermore, the data support the hypothesized negative relationship between social problem-solving skills and the aforementioned variables, including suicidal ideation, parent–adolescent conflicts, anxiety, and depression. This inverse relationship suggests that higher levels of social problem-solving skills are associated with lower levels of suicidal ideation, parent–adolescent conflicts, anxiety, and depression among adolescents. These findings underscore the potential protective role of social problem-solving skills in adolescent mental health and family dynamics.

The regression analysis examining the relationship between parent–adolescent conflict scores and suicidal ideation (Table 3) revealed a significant predictive effect only in the adolescent and peer intervention group (Group 2). No statistically significant predictive relationships were observed in the adolescent-only group or the adolescent-plus-parent group. Regarding anxiety and depression, the pre-test scores demonstrated statistically significant predictive effects on suicidal ideation across all three groups.

The results of the post-test correlation matrix (Table 4) indicate similar findings as pre-test. A positive and statistically significant correlation of anxiety with depression, suicidal ideation, and parent–adolescent conflicts. It negatively correlated with social problem solving. When considering the depression and anxiety scale, it is positively and significantly correlated with the anxiety scale, depression scale, suicidal ideation scale, and parent–adolescent conflict scale. It negatively and statistically significantly correlated with social problem-solving scales.

Table 5 indicates that there are, in general, no between-group differences between the three intervention groups, meaning that the three groups were on the same baseline before the intervention. The only scale showing a statistically significant difference is depression level, in which the score for the adolescents’ group is one point higher than the adolescent and peers’ group and two points higher than the adolescent and parent groups. In general, that is acceptable.

### 3.2. Comparing the Three Interventions at Pre-Test and Post-Test

The analysis of the intervention outcomes (Table 6) reveals a consistent reduction in adolescents’ anxiety, depression, suicidal ideation, and parent–adolescent conflict across all three intervention types. Notably, the adolescent-only group demonstrated the most substantial reductions in these measures post-intervention, while the adolescent-plus-peers group showed comparatively smaller reductions.

Repeated measures analysis of variance (RM-ANOVA) was employed to assess both within-group and between-group differences across all measurements. As indicated in Table 6, anxiety level was reduced after adolescents joined any of the three intervention groups. The adolescents only group (Group A) reported the highest reduction in mean scores before and after the intervention (mean difference of 2.4, 1.1, and 0.2 for the adolescent group (Group A), adolescent and peers (Group B) group, and adolescent and parents group (Group C), respectively). Concerning depression symptoms, Table 6 shows that depression levels decreased in the three interventions (mean differences in groups A, B, and C of −3.7, −2.2, and −1.7, respectively). The group mean differences reveal a higher difference in Group A than in Group B and Group C. Statistically significant within-group differences were observed for anxiety (F(1, 100) = 10.7, *p* = 0.01), depression (F(1, 97) = 51.8, *p* = 0.01), combined anxiety and depression (F(1, 97) = 32.9, *p* = 0.01), and suicidal ideation (F(1, 98) = 21.4, *p* = 0.01). These results indicate that all three interventions effectively reduced these symptoms. However, no statistically significant between-group differences were found for any of these measures.

For suicidal ideation, Table 6 shows that a reduction in suicidal ideation occurred following all three interventions (mean difference in groups A, B, and C of −4.8, −5.1, and −0.3, respectively). There was a statistically significant within-group difference (F(1, 98) = 21.4, *p* = 0.01), meaning that a reduction in the suicidal ideation score was statistically significant. Concerning the between-group differences, RM-ANOVA indicated there was no statistically significant difference (F(2, 98) = 0.16, *p* = 0.86).

Regarding parent–adolescent conflict, while reductions were observed across all interventions, the within-group differences did not reach statistical significance (F(1, 98) = 30.6, *p* = 0.08), indicating that the observed reductions may be attributable to chance rather than the interventions. Statistical testing also indicated no between-group differences (F(2, 98) = 0.21, *p* = 0.82), showing no statistically significant difference between the three interventions.

In conclusion, the study demonstrates statistically significant reductions in anxiety, depression, combined anxiety and depression, and suicidal ideation across all three intervention types. The absence of significant between-group differences suggests that the interventions are beneficial for adolescents, regardless of the specific approach employed. These findings contribute valuable insights to the field of adolescent mental health interventions, highlighting the potential promise of diverse approaches in addressing key psychological risk factors associated with adolescent suicide.

## 4. Discussion

The correlation matrix indicates a statistically significant correlation of adolescent suicidal ideation with the parent-adolescent conflict scale, anxiety, and depression scale, suggesting the importance of family relationships in adolescent mental health. High levels of conflict within the parent–adolescent conflicts may contribute to feelings of hopelessness and distress, which have the possibility of leading to suicidal thoughts. This finding is in general consistent with other studies indicating adolescents experiencing family conflicts were at higher risk of suicidal intention [35]. The statistical analyses comparing pre- and post-intervention results in this study provide evidence for the effectiveness of all three types of interventions in significantly reducing anxiety, depression, combined anxiety and depression scores, and suicidal ideation among adolescent participants. The findings of a reduction in suicidal ideation scores among participants were generally consistent with previous research on school-based suicide prevention programs, which indicates a statistically significant decrease in suicidal thoughts and behaviors in earlier studies [21]. These findings represent a substantial contribution to the planning, development and deployment of suicide prevention programs in secondary schools, addressing key risk factors that have been consistently identified in both local and international research as major contributors to adolescent suicide. The study provides information that adolescents exhibiting depression and suicidal ideation can derive some benefits from participating in CBT intervention programs. Notably, the interventions demonstrated promising outcomes across all three modalities: adolescent-only groups, adolescent–peer groups, and adolescent-parent groups, with the adolescent-only group showing the most pronounced reductions in depression, anxiety, and suicidal ideation. The statistically significant changes observed before and after the intervention suggest that these improvements can be partially attributed to the interventions, as there was no control group in this study. While reductions in parent–adolescent conflict were observed, the changes did not reach statistical significance. The observed statistical non-significance in certain intervention groups may be attributed to the differential predictive capacity of the pre-test parent–adolescent conflict scores on suicidal ideation across the various intervention modalities. Specifically, the regression analysis revealed no statistically significant predictive relationship between parent–adolescent conflict and suicidal ideation in both the adolescent-only group and the adolescent-plus-parent group. In contrast, a statistically significant association was demonstrated in the adolescent and peer intervention groups. The reduction in score of measurement may partly be attributed to their focus on enhancing participants’ awareness of automatic thoughts in response to stressors, a cognitive process often associated with depressive and anxious symptomatology. The group sessions trained participants to identify and reframe these automatic thoughts, fostering more adaptive and positive cognitive patterns. These findings underscore the complexity of the relationship between family conflict and suicidal thoughts in adolescents and highlight the importance of considering intervention-specific factors when examining such associations. Further research is warranted to elucidate the mechanisms underlying these differential effects and to inform the development of targeted, context-specific interventions for adolescent suicide prevention.

## 5. Limitations

While this study offers valuable insights into the possible outcomes of various adolescent suicide prevention interventions, several methodological limitations warrant consideration. The relatively small sample size (n = 105) drawn from five secondary schools in Hong Kong may constrain the generalizability of findings to the broader adolescent population. The absence of a non-intervention control group limits the ability to definitively attribute observed changes solely to the interventions, as natural maturation or extraneous factors may have influenced outcomes. The statistically significant reduction in those measurements following the intervention may be attributed to the phenomenon of “regression to the mean”, making it difficult to solely attribute the results to the intervention itself. The study’s design lacked long-term follow-up assessments, precluding an understanding of the interventions’ sustained effects. Reliance on self-report measures to assess anxiety, depression, and suicidal ideation introduces potential biases stemming from social desirability or limited self-awareness among participants. The study’s focus on Hong Kong secondary schools may restrict the applicability of findings to diverse cultural contexts or educational systems. Despite implementing three distinct intervention types, the absence of statistically significant between-group differences limits the ability to discern the most effective approach. The voluntary nature of participation may have introduced selection bias, potentially affecting the sample’s representativeness. The relatively small sample size, lack of a control group, short-term follow-up, and potential selection bias may limit the generalizability of the findings. Additionally, the reliance on self-report measures and the study’s focus on Hong Kong secondary schools may restrict the broader applicability of the results. Addressing these limitations in future research would enhance the robustness and generalizability of findings in the critical domain of adolescent suicide prevention.

## 6. Implications

The findings highlight the potential of applying cognitive-behavioral therapy (CBT) within school-based suicide prevention programs in Hong Kong. Additionally, this research underscores the need for social work and counseling practitioners in secondary school settings to focus more on supporting high-risk students through group work. It emphasizes the importance of addressing peer influence and family dynamics in the design and implementation of these programs, ensuring a holistic approach to mental health support for adolescents.

## Figures and Tables

**Table 1:** Basic demographic data.

	Group A	Group B	Group C	Total
	N = 49	N = 26	N = 30	
Mean Age	15.03	14.21	15.18	N/A
Male	20	16	6	42
Female	29	10	24	63
Years of residence in HK	12.29	13.32	13.37	*p* = 0.30
Place of birth	*n* (%)	*n* (%)	*n* (%)	*p* = 0.38
Hong Kong	40 (81.6%)	25 (84.6%)	24 (80.0%)	89 (84.8%)
Mainland China	8 (16.3%)	1 (3.8%)	5 (16.7%)	14 (13.3%)
Other	1 (2.0%)	0	1 (3.3%)	2 (1.9%)
Chronic Illness				*p* = 0.43
No	19 (38.8%)	22 (84.6%)	20 (66.7%)	61 (58.1%)
Yes	7 (14.3%)	3 (11.5%)	5 (16.7%)	15 (14.3%)
Missing	23 (46.9%)	1 (3.8%)	5 (16.7%)	29 (27.6)
No. of Siblings	1.79	1.64	1.88	*p* = 0.58
Parents’ Marital Status				*p* = 0.17
Married	31 (63.3%)	16 (61.5%)	20 (66.7%)	67 (63.8%)
Remarriage	0	0	1 (3.3%)	1 (1.0%)
Divorced/separated	6 (12.3%)	5 (19.2%)	3 (10.0%)	14 (13.4%)
Cohabitation	1 (2.0%)	4 (15.4%)	0	5 (4.8%)
Widowed	1 (2.0%)	0	0	1 (1.0%)
Missing	10 (20.4%)	1 (3.8%)	6 (20.0%)	17 (16.2%)
Cohabitation with				
Father	29 (59.2%)	19 (73.1%)	19 (63.3%)	67 (63.8%)
Mother	36 (73.5%)	24 (92.3%)	23 (76.6%)	83 (79.0%)
Grandparent(s)	1 (2%)	4 (15.4%)	3 (20.0%)	11 (10.5%)
Relative(s)/others	2 (4.1%)	2 (7.6%)	1 (3.3%)	5 (4.8%)
Sibling(s)	17 (34.7%)	14 (53.8%)	14 (46.7%)	45 (42.9%)
Employment Status—Father				*p* = 0.91
	37 (75.5%)	20 (76.9%)	19 (63.3%)	76 (72.4%)
Full Time	4 (8.2%)	1 (3.8%)	1 (3.3%)	6 (5.7%)
Part Time (~22 h)	2 (4.1%)	0	0	2 (1.9%)
Daily/Hourly Paid (<22 h)	2 (4.1%)			
Housework	0	0	1 (3.4%)	1 (1.0%)
Unemployed	2 (4.1%)	1 (3.8%)	0	4 (3.8%)
Other	3 (6.1%)	3 (11.5%)	1 (3.3%)	7 (6.7%)
Missing	1 (2.0%)	1 (3.8%)	7 (23.3%)	9 (8.6%)
Educational Level—Father				*p* = 0.87
No formal education	1 (2.0%)	0	0	1 (1.0%)
Primary school	3 (6.1%)	3 (11.5%)	2 (6.7%)	8 (7.6%)
Form 1 to 3	9 (18.4%)	8 (30.8%)	4 (13.3%)	21 (20.0%)
Form 4 to 5	14 (28.6%)	4 (15.4%)	7 (23.3%)	25 (23.8%)
Form 6 to 7	11 (22.4%)	7 (26.9%)	7 (23.3%)	25 (23.8%)
Post-secondary	9 (18.4%)	3 (11.5%)	3 (10.0%)	15 (14.3%)
Missing	2 (4.1%)	1 (3.8%)	7 (23.3%)	10 (9.5%)
Employment Status—Mother				*p* = 0.02 *
Full Time	22 (44.9%)	12 (46.2%)	16 (53.3%)	50 (47.6%)
Part Time (~22 h)	6 (12.2%)	3 (11.5%)	3 (10.0%)	12 (11.4%)
Daily/Hourly Paid (<22 h)	0	3 (11.5%)	4 (13.3%)	7 (6.7%)
Housework	17 (34.7%)	6 (23.1%)	2 (6.7%)	25 (23.8%)
Unemployed	4 (8.2%)	1 (3.8%)	0	5 (4.8%)
Other	0	0	0	
Missing	0	1 (3.8%)	5 (16.7%)	6 (5.7%)
Educational Level—Mother				*p* = 0.64
No formal education	1 (2.0%)	1 (3.8%)	0	2 (1.9%)
Primary school	8 (16.3%)	2 (7.7%)	2 (6.7%)	12 (11.4%)
Form 1 to 3	11 (22.4%)	10 (38.5%)	6 (20.0%	27 (25.7%)
Form 4 to 5	11 (22.4%)	2 (7.7%)	5 (16.7%)	18 (17.1%)
Form 6 to 7	11 (22.4%)	5 (19.2%)	7 (23.3%)	23 (21.9%)
Post-secondary	5 (10.2%)	5 (19.2%)	4 (13.3%)	14 (13.3%)
Missing	2 (4.1%)	1 (3.8%)	6 (20.0%)	9 (8.6%)
Monthly Household Income (HK$)				*p* = 0.80
10,000 or below	5 (10.2%)	2 (7.7%)	1 (3.3%)	8 (7.6%)
10,001 to 30,000	23 (46.9%)	13 (50.5%)	10 (33.3%)	46 (43.8%)
30,001 to 50,000	9 (18.4%)	6 (23.1%)	6 (20.0%)	21 (20.0%)
50,001 or above	5 (10.2%)	4 (15.4%)	6 (20.0%)	15 (14.3%)
Missing	7 (14.3%)	1 (3.8%)	7 (23.3%)	15 (14.3%)
CSSA (Family living on financial assistance from the government)				*p* = 0.29
No	40 (81.6%)	24 (92.3%)	23 (76.7%)	87 (82.9%)
Yes	8 (16.3%)	1 (3.8%)	2 (6.7%)	11 (10.5%)
Missing	1 (2.0%)	1 (3.8%)	(16.7%)	(6.7%)

* *p* < 0.05.

**Table 2:** Correlation matrix of different variables before intervention.

	Anx	Dep	A&D	SIS	PAC
Anxiety		0.388 **	0.866 **	0.477 **	0.211 *
Depression	0.388 **		0.797 **	0.366 **	0.381 **
Anxiety Depression	0.866 **	0.797 **		0.517 **	0.343 **
Suicidal Ideation	0.477 **	0.366 **	0.517 **		0.331 **
Parent–Adolescent Conflict	0.211 **	0.381 **	0.343 **	0.331 **	

* *p* < 0.05; ** *p* < 0.001. Anx = anxiety; Dep = depression; A&D = anxiety and depression; SIS = suicidal ideation; PAC = parent–adolescent conflict.

**Table 3:** Regression analysis between suicide ideation and other variables at pre-test.

	R^2^	F	B	β	*t*
Parent–Adolescent Conflict					
Group 1: Adolescent-only group	0.073	3.381	0.42	0.27	1.893
Group 2: Adolescent and peer group	0.399	14.57	0.625	0.631	3.818 **
Group 3: Adolescent plus parent group	0.041	1.160	0.243	0.203	1.077
Anxiety and Depression					
Group 1: Adolescent-only group	0.183	10.06	0.579	0.427	3.171 *
Group 2: Adolescent and peer group	0.185	4.990	0.569	0.430	2.236 *
Group 3: Adolescent plus parent group	0.450	21.30	0.619	0.671	4.62 **

* *p* < 0.05, ** *p* < 0.001.

**Table 4:** Correlation matrix of different variables after intervention.

	Anx	Dep	A&D	SIS	PAC
Anxiety		0.568 **	0.920 **	0.558 **	0.885 **
Depression	0.568 **		0.845 **	0.374 **	0.865 *
Anxiety Depression	0.920 **	0.845 **		0.542 **	0.897 **
Suicidal Ideation	0.558 **	0.374 **	0.552 **		0.808 **
Parent–Adolescent Conflict	0.885 **	0.865 **	0.897 *	0.808 *	

* *p* < 0.05; ** *p* < 0.001. Anx = anxiety; Dep = depression; A&D = anxiety and depression; SIS = suicidal ideation; PAC = parent–adolescent conflict.

**Table 5:** Pre-score comparison of the three interventions.

	Group A		Group B		Group C		
	Adolescents *N* = 49		Adolescents-Plus-Peers Group *N* = 26		Adolescents-Plus-Parents Group *N* = 30		
	Mean	SD	Mean	SD	Mean	SD	
Anxiety	11.1	4.2	10.7	3.1	9.8	4.0	*p* = 0.24
Depression	10.7	3.1	9.7	3.0	8.6	3.8	*p* = 0.03 *
Anxiety Depression	21.7	5.8	19.7	4.7	18.4	7.5	*p* = 0.06
Suicidal Ideation	30.3	8.0	29.6	6.1	27.2	7.3	*p* = 0.21
Parent–Adolescent Conflict	9.7	5.2	8.7	6.0	8.0	6.1	*p* = 0.45

* *p* < 0.05.

**Table 6:** Interaction effects: within-group and between-group differences RM-ANOVA.

	Group A				Group B				Group C									
	Adolescents *N* = 49				Adolescents-Plus-Peers Group *N* = 26				Adolescents-Plus-Parents Group *N* = 30				Within-Group Differences			Between-Group Differences		
	Pre		Post		Pre		Post		Pre		Post							
	M	SD	M	SD	M	SD	M	SD	M	SD	M	SD	*df*	*F*	*p*	*df*	*F*	*p*
Anxiety	11.1	4.2	8.7	4.1	9.7	3.4	8.6	4.2	9.8	4.0	9.6	3.9	1	10.7	0.01 *	2	0.19	0.83
Depression	10.7	3.1	7.0	2.7	9.7	3.0	7.5	3.5	8.6	3.8	6.9	3.0	1	51.8	0.01 *	2	2.2	0.12
Anxiety Depression	21.7	5.8	15.7	5.8	19.6	4.7	16.2	7.2	18.4	7.5	16.0	6.3	1	34.9	0.01 *	2	0.75	0.47
Suicidal Ideation	30.3	8.0	25.5	7.3	29.6	6.1	24.5	6.9	27.2	7.3	26.9	7.8	1	21.4	0.01 *	2	0.16	0.86
Parent- Adolescent Conflict	9.7	5.2	7.9	5.8	8.7	6.0	7.7	6.7	8.0	6.1	8.1	5.3	1	30.6	0.08	2	0.21	0.82

M = mean; SD = standard deviation; df = degree of freedom; * *p* < 0.01.

## Data Availability

The original contributions presented in the study are included in the article, further inquiries can be directed to the corresponding author.
